# Access to capped RNAs by chemical ligation†

**DOI:** 10.1039/d4cb00165f

**Published:** 2024-09-13

**Authors:** Karolina Bartosik, Ronald Micura

**Affiliations:** a Institute of Organic Chemistry, Center for Molecular Biosciences Innsbruck, University of Innsbruck, Innrain 80-82 6020 Innsbruck Austria ronald.micura@uibk.ac.at

## Abstract

A distinctive feature of eukaryotic mRNAs is the presence of a cap structure at the 5′ end. The typical cap consists of 7-methylguanosine linked to the first transcribed nucleotide through a 5′,5′-triphosphate bridge. It plays a key role in many processes in eukaryotic cells, including splicing, intracellular transport, initiation of translation and turnover. Synthetic capped oligonucleotides have served as useful tools for elucidating these physiological processes. In addition, cap mimics with artificial modifications are of interest for the design of mRNA-based therapeutics and vaccines. While the short cap mimics can be obtained by chemical synthesis, the preparation of capped analogs of mRNA length is still challenging and requires templated enzymatic ligation of synthetic RNA fragments. To increase the availability of capped mRNA analogs, we present here a practical and non-templated approach based on the use of click ligation resulting in RNAs bearing a single triazole linkage within the oligo-phosphate backbone. Capped RNA fragments with up to 81 nucleotides in length have thus been obtained in nanomolar yields and are in demand for biochemical, spectroscopic or structural studies.

## Abstract 2

A practical approach to generate mRNA 5′-fragments with clicked triazole linkages in the backbone is presented.

## Graphical abstract

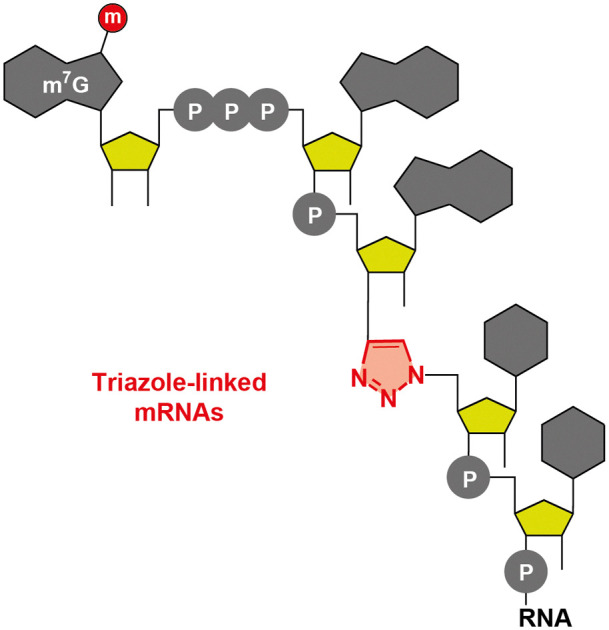

## Introduction

**Fig. 1:**
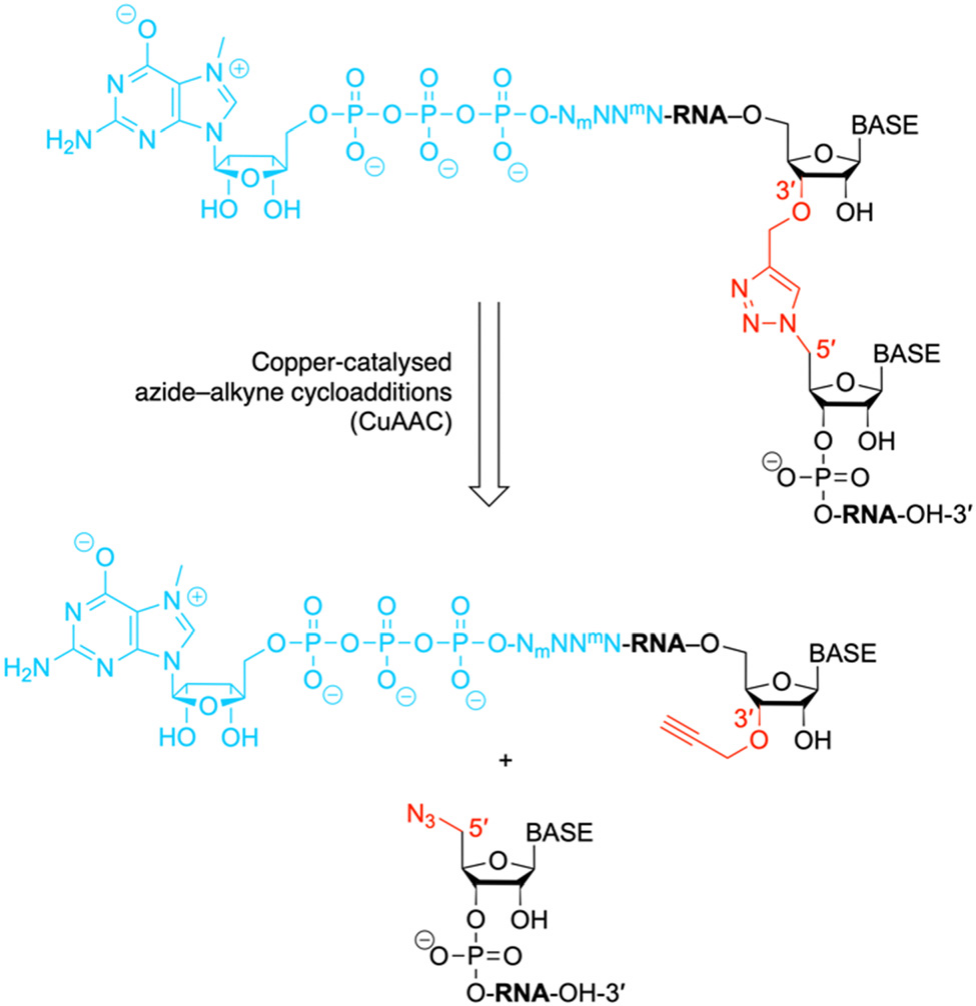
The chemical structure shows an exemplary mRNA with a biocompatible triazole linkage (TrzB)^10^ as phosphate backbone replacement. The present work describes a chemical ligation approach to generate such target compounds. N, nucleotide; N_m_ and ^m^N, modified nucleotides.

Messenger RNAs (mRNAs) of eukaryotes contain a characteristic 7-methylguanosine (m^7^G)-5′-ppp moiety at their 5′-end, the so-called “cap”.^1,2^ In addition, the terminus of mRNA is often heavily methylated at the first four nucleosides, such as in trypanosomatids.^3,4^ Other aspects of modification include artificially altered mRNA caps and cap mimics, such as those tailored for mRNA vaccines.^5^ All of these modifications can critically affect translation efficiency, nuclear stability, and binding affinity to the many enzymes that interact with the cap in the cell.^1^ While the synthesis of modified di- and trinucleotide caps is a domain of synthetic organic chemistry,^6–9^ the length of mRNAs is typically well beyond the scope of chemical synthesis. Here, we demonstrate that current size limitations of synthetic capped 5′-mRNA can be overcome by chemical ligation (applying click chemistry)^10^ to generate RNA with single units of triazole backbone linkages (Fig. 1). This approach provides an alternative to synthetic mRNA obtained by enzymatic ligation of chemically synthesized RNA fragments, using T4 DNA ligases and splint oligos.^11^ Capped RNA fragments from 6 to 81 nucleotides in length, accessible by this approach in nanomolar yields, are in demand for biochemical, spectroscopic or structural studies.

Moreover, the approach presented here is an important step towards efficient all-chemical synthesis of mRNAs without the use of *in vitro* transcription (IVT) for RNA synthesis, which usually results in a significant fraction of uncapped transcripts and additionally in double-stranded RNA by-products that cause an undesired immunogenic response.^2^ mRNA generated by IVT requires intensive purification,^2,12^ and therefore alternatives—such as the one presented here—for all-chemical mRNA synthesis are of current interest.^13,14^ It is worth mentioning that first concepts combining chemical cap synthesis, chemical RNA synthesis and click bioconjugation to obtain short mRNA fragments were described by the Jemielity group and date back to 2017.^15^

## Results and discussion

### Established approaches to synthesize short RNAs with caps

The most common strategy to synthesize RNA caps in solution is based on the reaction between an activated derivative of 7-methylguanosine 5′-diphosphate (m^7^GDP) with a 5′-phosphorylated oligoribonucleotide in the presence of metal ions. Several phosphate activating groups (phenylthiol,^16^ methoxyphenylthio,^17,18^ 5-chloro-8-quinolyl,^19^ and imidazolide,^20–24^ in combination with diverse divalent metal catalysts (MgCl_2_, MnCl_2_, CaCl_2_, CdCl_2_, CuCl_2_ and ZnCl_2_),^20^ have been described. The most practical and widely applied conditions employ the imidazolide group and zinc chloride under anhydrous conditions, providing capped oligoribonucleotides in good to sufficient yields. One drawback is, however, the limitation to rather short (up to 4 nt) RNAs.^21,24^ Recently, Abe and coworkers developed a chemical method allowing the synthesis of longer sequences bearing cap modification.^14^ In this method, the fully deprotected 5′-phosphorylated RNAs were reacted with Im-m^7^GDP using 1-methylimidazole as an activator and DMSO as a solvent. It enabled quantitative preparation of 107 nt capped RNAs within 3 h; a huge excess of capping reagent was required and the RNAs were obtained in picomol amounts only.

The instability of m^7^G under acidic (depurination) and basic conditions (opening of the m^7^G imidazole ring) makes the chemical solid-phase synthesis of cap RNAs challenging.^25^ For this reason, some of the methods utilize weak acid-labile^26^ or disulfide linkers^27^ between the RNA chain and solid support instead of standard base-labile linker. These methods include the coupling of imidazolide derivative of m^7^G mono- or di-phosphate with the support-bound 5′-di- or 5′-mono-phosphorylated RNA, respectively. After release from the support, the yield of capped RNAs was rather low. To increase the efficiency of capped RNA synthesis, Debart and coworkers developed a method that combined solid-phase RNA synthesis and enzymatic *N*7 methylation of the guanine moiety.^28^ In more detail, the short RNAs (from 4 to 18 nt), prepared on solid support, were phosphorylated at the terminal 5′-OH, activated by imidazole, and then reacted with GDP. The Gppp-RNAs were purified on HPLC before being enzymatically methylated using human (guanine-*N*7)-methyltransferase. Recently, the same group reported an alternative approach exploiting m^7^GDP attachment to fully protected resin-bound RNAs.^13^ The previously employed PivOM-protected monomers^28^ were replaced by more labile PrOM-protected building blocks, allowing the capped RNAs to be deprotected under mild conditions that prevented m^7^G degradation. The authors synthesized capped oligoribonucleotides of different sequences and lengths (up to 28 nt).

### Intended concept to overcome size limitations in the synthesis of capped RNA by chemical ligation

The ligation of short chemically synthesized RNA strands by copper-catalyzed azide–alkyne cycloadditions (CuAAC coupling) was previously shown to offer a valid and efficient strategy for the generation of large, chemically modified RNA constructs (*e.g.* ribozymes).^10,29^ In particular, the TrzB linkage (Fig. 1) – originally introduced by Brown and coworkers – turned out to be useful because it displays high biocompatibility.^10,29^ This linkage was recently also used for the generation of pools of single guide RNAs (sgRNAs) in the emerging field of CRISPR-Cas (clustered regularly interspaced palindromic repeats) gene editing.^30^

Encouraged by these early studies, we intended to explore the TrzB linkage in the context of mRNA synthesis (Fig. 1). We exemplify our ligation endeavours for cap-4 spliced leader (SL) RNA of Trypanosomatid parasites.^3,4,11^ In short, a hexanucleotide m^7^G-5′-ppp-5′-(m_2_^6^Am)(Am)(Cm)(m^3^Um)(3′-*O*-propargyl-A) offering an alkyne group at the 3′ end, is generated and effectively ligated to a chemically synthesized RNA bearing an azido group at the 5′ end, following untemplated cooper-catalyzed azide–alkyne cycloaddition (CuAAC) chemistry (Fig. 1). The resultant cap4 SL RNAs of about 19 to 81 nts in length, containing an artificial triazole backbone at the site of ligation, provide valuable probes for ongoing and future biochemical and structural studies (cryo-EM and X-ray crystallography) of these RNAs in complex with enzymes recognizing mRNAs with the short hypermethylated cap4 modality.

### Synthesis of 3′-alkyne m^7^G-5′ppp-5′-oligonucleotides

**Fig. 2:**
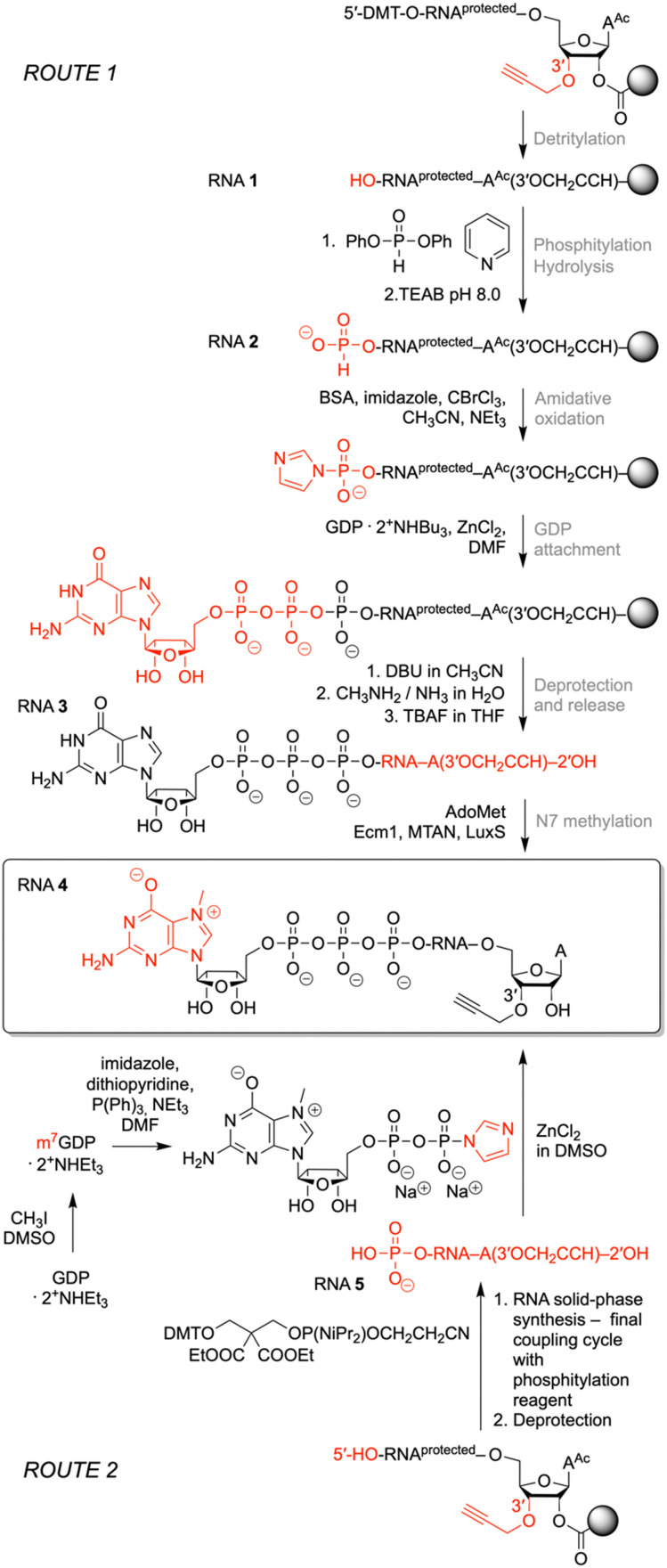
Two routes for the preparation of m^7^Gppp-RNA 3′-alkyne: solid-phase synthesis of Gppp-RNA 3′-alkyne followed by enzymatic *N*7 methylation (top), and alternatively, the synthesis of 5′-p-RNA 3′-alkyne followed by reaction with Im-m^7^GDP (bottom). Reagents and conditions: ROUTE 1: detritylation: 3% dichloroacetic acid in Cl_2_HCCHCl_2_; phosphitylation: 0.1 M diphenyl phosphite in py, rt, 10 min; hydrolysis: 0.1 M TEAB in H_2_O/ACN (6/4 v/v), rt, 20 min; amidative oxidation: imidazole, *N*,*O*-bis(trimethylsilyl)acetamide, CBrCl_3_, TEA, ACN, rt, 1 h; *GDP* attachment: 0.28 M GDP· 2Bu_3_NH^+^ and 0.5 M ZnCl_2_ in DMF, rt, 16 h; deprotection and release: (i) 1.0 M DBU/ACN, rt, 5 min, (ii) 40% aq MeNH_2_/30% aq NH_3_ (1/1 v/v), 40 °C, 4 h, (iii) 1 M TBAF/THF, 37 °C, 16 h; *N*7 methylation: phosphate buffer (1.5 M NaCl, 200 mM Na_2_HPO_4_, pH 7.4), AdoMet, Ecm1 methyltransferase, MTAN nucleosidase, LuxS lyase, 37 °C, 1 h. ROUTE 2: GDP methylation: CH_3_I/DMSO, rt, 3 h; activation: imidazole, 2,2′-dithiopyridine, Ph_3_P, TEA, DMF, rt, 24 h; cap installation: Im-m^7^GDP/DMSO, ZnCl_2_, rt, 24 h or 55 °C, 3 h.

**Fig. 3:**
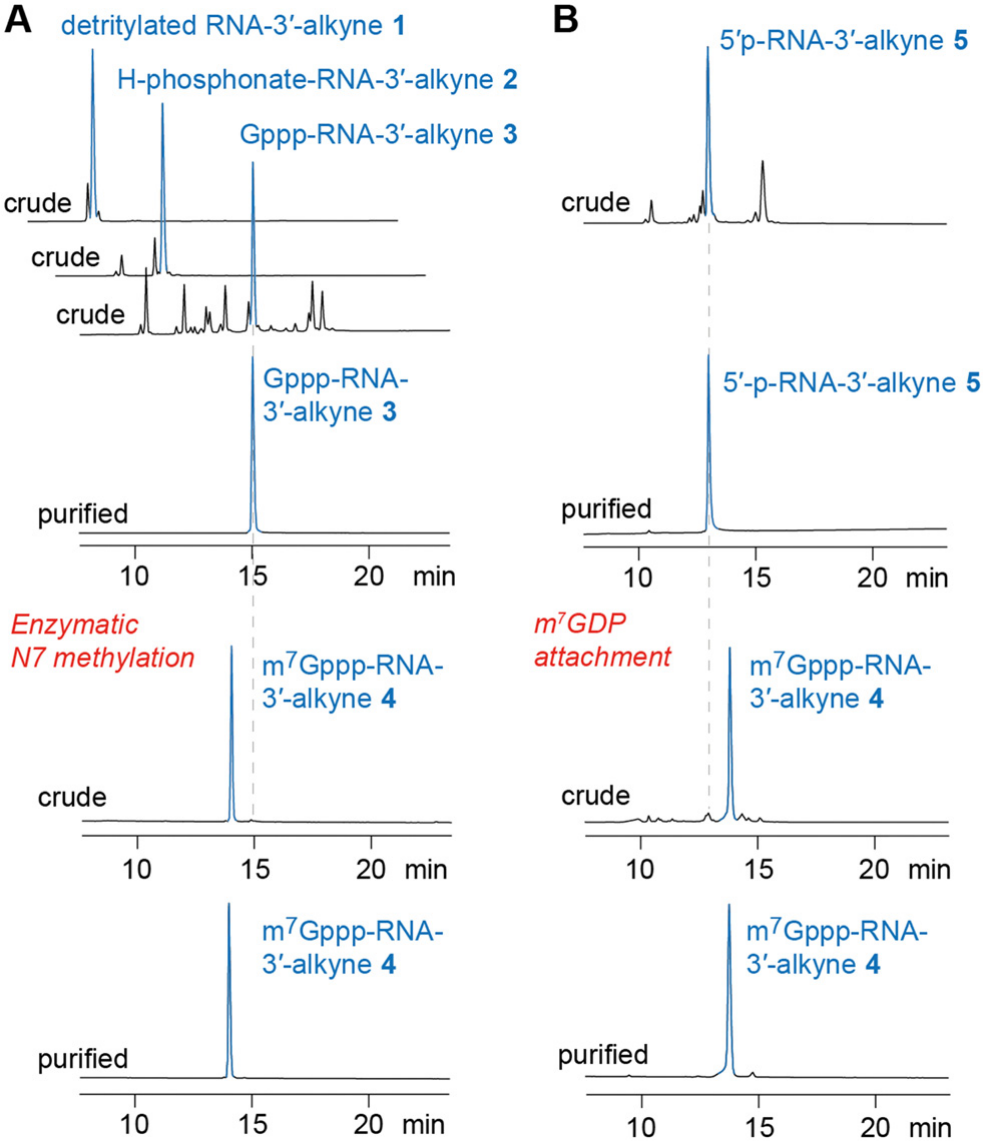
HPLC analysis of m^7^Gppp-RNA 3′-alkyne preparations (exemplified for m^7^G-5′-ppp-5′-(m_2_^6^Am)(Am)(Cm)(m^3^Um)(3′-*O*-propargyl-A, RNA 4). (A) Path for chemoenzymatic synthesis. (B) Path for all-chemical synthesis (for details see Fig. 2 and Table 1 and the ESI†).

To prepare short m^7^G-ppp-oligoribonucleotides bearing alkyne functionality at the 3′ end, two alternative routes were devised (Fig. 2). The first one followed the procedure that we previously applied to obtain a 39 nt cap-4 SL RNA fragment of *Trypanosoma cruzi*.^11^ The novel aspect here was the application of a (commercially available) CPG solid support that provided the terminal 3′-*O*-propargyl nucleoside of interest. The four methylated nucleosides m_2_^6^Am, Am, Cm, and m^3^Um were assembled into the sequence of 5′-DMT-*O*-(m_2_^6^Am)(Am)(Cm)-(m^3^Um)(3′-*O*-propargyl-A) by standard phosphoramidite chemistry (Fig. 2 and 3 and Table 1)). After 5′-*O* detritylation, the 5′ hydroxyl group of the CPG-bound protected RNA (RNA 1) was converted into the *H*-phosphonate RNA derivative by treatment with diphenyl phosphite and subsequent hydrolysis with triethylammonium bicarbonate buffer (RNA 2) (Fig. 2, 3 and Table 1). Then, oxidation and activation resulted in the corresponding 5′-phosphoroimidazolide RNA, that was reacted with the tri-butylammonium salt of GDP in DMF in the presence of ZnCl_2_ (RNA 3) (Fig. 2, 3 and Table 1). All these steps were performed while the protected RNA was bound to the support, thus allowing the removal of excess reagents by simple washing which makes the synthesis convenient. The final release from the support and deprotection yielded the crude G-5′-ppp-5′-(m_2_^6^Am)(Am)(Cm)(m^3^Um)(3′-*O*-propargyl-A), which was purified by anion exchange (AE) HPLC (RNA 3) (Fig. 2, 3 and Table 1 and Fig. S1, ESI†). Methylation of guanine at position *N*7 was performed enzymatically using Ecm1 methyltransferase in the presence of AdoMet as the methyl donor (Fig. 2 and 3). The conversion of G-5′-ppp-5′-(m_2_^6^Am)(Am)(Cm)(m^3^Um)(3′-*O*-propargyl-A) (RNA 3) into m^7^G-5′-ppp-5′-(m_2_^6^Am)(Am)(Cm)(m^3^Um)(3′-*O*-propargyl-A) (RNA 4) proceeded very clean and quantitative in less than 1 h of incubation at 37 °C (Fig. 2 and 3 and Table 1 and Fig. S1, ESI†).

**Table 1:** Sequences and characterization data of RNAs for the synthesis of triazole-linked cap4 analogs

RNA No.	Sequence	nt	Scale	Isolated amount^a^ nmol	m.w. (calc.) [amu]	m.w. (found) [amu]
Short capped RNAs (Route 1)						
1	5′-HO-(m_2_^6^Am)(Am)(Cm)(m^3^Um)A-3′-alkyne^b^	5	—	—	1673.3	1672.7
2	5′-Hp-(m_2_^6^Am)(Am)(Cm)(m^3^Um)A-3’-alkyne^b^^c^	5	—	—	1737.2	1736.6
3	Gppp-(m_2_^6^Am)(Am)(Cm)(m^3^Um)A-3′-alkyne	6	1.0 μmol	75	2178.4	2178.2
4	m^7^Gppp-(m_2_^6^Am)(Am)(Cm)(m^3^Um)A-3′-alkyne	6	20 nmol	18	2192.4	2191.9
Short capped RNAs (Route 2)						
5	5′-p-(m_2_^6^Am)(Am)(Cm)(m^3^Um)A-3′-alkyne	5	1.0 μmol	140	1753.2	1752.6
4	m^7^Gppp-(m_2_^6^Am)(Am)(Cm)(m^3^Um)A-3′-alkyne	6	20 nmol	12	2192.4	2191.7
5′-azide RNAs						
6	5′-N_3_-ACGCUAUUAUUGA-OH-3′	13	1.0 μmol	114	4111.5	4111.3
7	5′-N_3_-ACGCUAUUAUUGAUACAGUUUCUGU-ACUAUAUUG-OH-3′	34	1.0 μmol	80	10 770.4	10 770.4
Triazole-linked (TrzB) RNAs						
8	m^7^Gppp-(m_2_^6^Am)(Am)(Cm)(m^3^Um)A-TrzB-ACG-CUAUUAUUGA-OH-3′	19	5 nmol	3.0	6303.9	6304.1
9	m^7^Gppp-(m_2_^6^Am)(Am)(Cm)(m^3^Um)A-TrzB-ACG-CUAUUAUUGAUACAGUUUCUGUACUAUA-UUG-OH-3′	40	5 nmol	2.5	12 962.9	12 963.1
12	m^7^Gppp-(m_2_^6^Am)(Am)(Cm)(m^3^Um)A-TrzB-ACGCUAUUAUUAGAACAGUUUCUGUACUAUAUUGGUAUGAGAAGCUCCCAGUAGC-OH-3′	61	5 nmol	1.5	19 783.0	19 782.5
13	m^7^Gppp-(m_2_^6^Am)(Am)(Cm)(m^3^Um)A-TrzB-ACGCUAUUAUUAGAACAGUUUCUGUACUAUAUUGGUAUGAGAAGCUCCCAGUAGCAGCUGGGCCAACACACGCAU-OH-3′	81	5 nmol	1.0	26 232.9	26 232.2

a Isolated amount refers to the amount of product that is produced from a single batch at the annotated scale and obtained after AE HPLC purification.

b For analytical purposes (HPLC), RNA from a small portion of the solid-support was deprotected and released to judge the quality of the individual reaction steps (see Fig. 3).

c Hp relates to H-phosphonate group.

For some laboratories, the use of enzymes might affect the attractiveness of the above approach. Therefore, we focused on an exclusively chemical access to short m^7^Gppp-RNA-3′-alkynes as alternative. We considered the reaction of 5′-phosphorylated RNA-3′-alkynes with the chemical capping agent 7-methylguanosine 5′-diphosphate imidazolide (Im-m^7^GDP)^21,24^ appropriate to generate the target m^7^G-5′-ppp-5′-(m_2_^6^Am)(Am)(Cm)(m^3^Um)(3′-*O*-propargyl-A) (RNA 4) (Fig. 2 and 3 and Table 1). Indeed, the 5′-phosphorylated pentamer 5′-p-(m_2_^6^Am)(Am)(Cm)(m^3^Um)(3′-*O*-propargyl-A) (RNA 5) (Fig. 2 and 3 and Table 1) was readily obtained by RNA solid-phase synthesis on the same solid support as used above and a typical building block for 5′-*O*-phosphorylation (3-DMTrO-2,2-bis(ethoxy-carbonyl)propyl 2-cyanoethyl *N*,*N*-diisopropylphosphor-amidite).^31,32^ Subsequently, the RNA was deprotected under standard conditions (AMA, 40 °C, 4h; 1 M TBAF, THF) and 5′-p-(m_2_^6^Am)(Am)(Cm)(m^3^Um)(3′-*O*-propargyl-A) (RNA 5) isolated by AE HPLC.

The synthesis of the Im-m^7^GDP was performed as described earlier^20,33^ whereby the triethylammonium salt of GDP was methylated using iodomethane, and the resulting *N*7-methylated GDP was precipitated before having been purified by RP-HPLC (Fig. 2). Then, m^7^GDP was converted to the imidazolide of the diphosphate using imidazole in the presence of an activation system including 2,2′dithiopyridine and triphenylphosphine. Im-m^7^GDP was isolated as the sodium salt.

For the capping reaction, we were inspired by the approach published by Jemielity and coworkers for the synthesis of cap2 trinucleotide.^23^ We performed the reaction between 5′-p-(m_2_^6^Am)(Am)(Cm)(m^3^Um)(3′-*O*-propargyl-A) (RNA 5) and Im-m^7^GDP at room temperature utilizing DMSO as a solvent and ZnCl_2_ as a catalyst. We increased the excess of reagents (to 50 equiv. of Im-m^7^GDP and to 25 equiv. of ZnCl_2_), which resulted in a shortening of the reaction time from 48 h to 24 h, obtaining the same yields (Fig. 2). Even more conveniently, the reaction time was further decreased (to 3 hours) at elevated temperature (55 °C). After AE HPLC purification, the integrity of m^7^G-5′-ppp-5′-(m_2_^6^Am)(Am)(Cm)(m^3^Um)-(3′-*O*-propargyl-A) (RNA 4) was confirmed by ESI MS analysis (Table 1 and Fig. S1, ESI†).

### Synthesis of 5′-azido-modified oligoribonucleotides

**Fig. 4:**
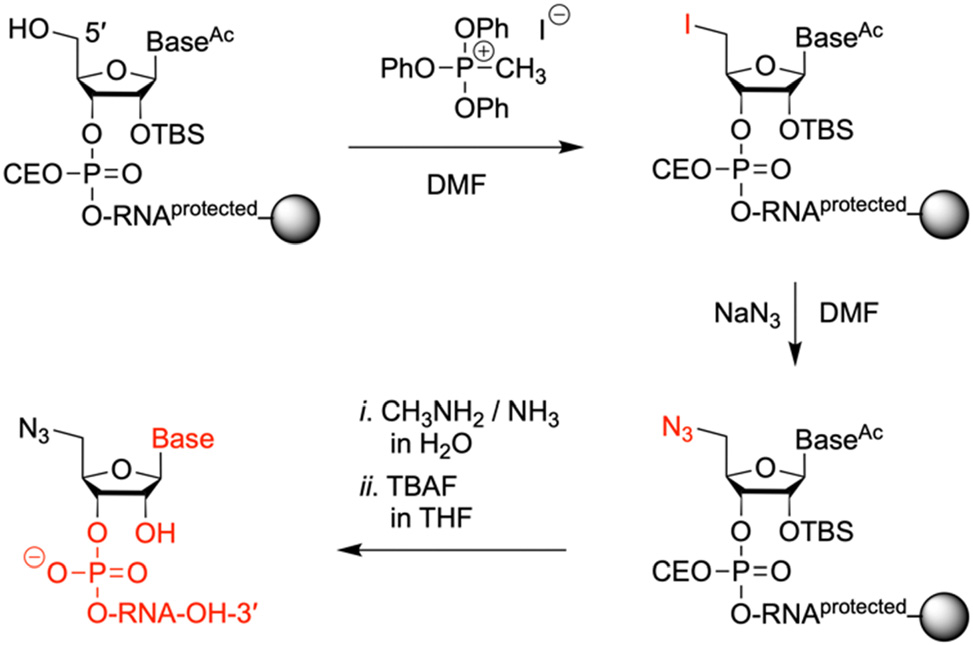
Solid-phase synthesis of 5′-azido modified oligoribonucleotides. Reagents and conditions: (1) 0.5 M MTPI/DMF, rt, 1 h; (2) sat. NaN_3_ solution in DMF, 55 °C, 5 h; (3) (i) 40% aq MeNH_2_/30% aq NH_3_ (1/1 v/v), 40 °C, 4 h, (ii) 1 M TBAF/THF, 37 °C, 16 h.

To prepare oligonucleotides bearing an azide moiety at the 5′ end, we oriented ourselves on earlier reported method, however, instead of relying on RNA solid-phase synthesis with 2′-*O*-silyl/*N*-tac-protected^29^ or 2′-*O*-PivOM/*N*-Pac-protected^15^ monomers, we implemented the standard 2′-*O*-silyl- and *N*-acetyl phosphoramidite building blocks. The azido group was incorporated on solid support by treating the 5′-OH deprotected RNA with methyltriphenoxyphosphonium iodide (MTPI), followed by incubation in a saturated solution of sodium azide in DMF (Fig. 4). After the azidination step, the RNAs were deprotected and cleaved from the support by using aqueous ammonia/methylamine solutions, and subsequently, tetrabutylammonium fluoride in THF. The crude RNAs were purified by ion-exchange chromatography (Table 1 and Fig. S1, ESI†), affording pure 5′-azido-functionalized oligoribo-nucleotides (RNA 6 and 7) in good overall yields (Table 1).

### Non-templated click ligation

**Fig. 5:**
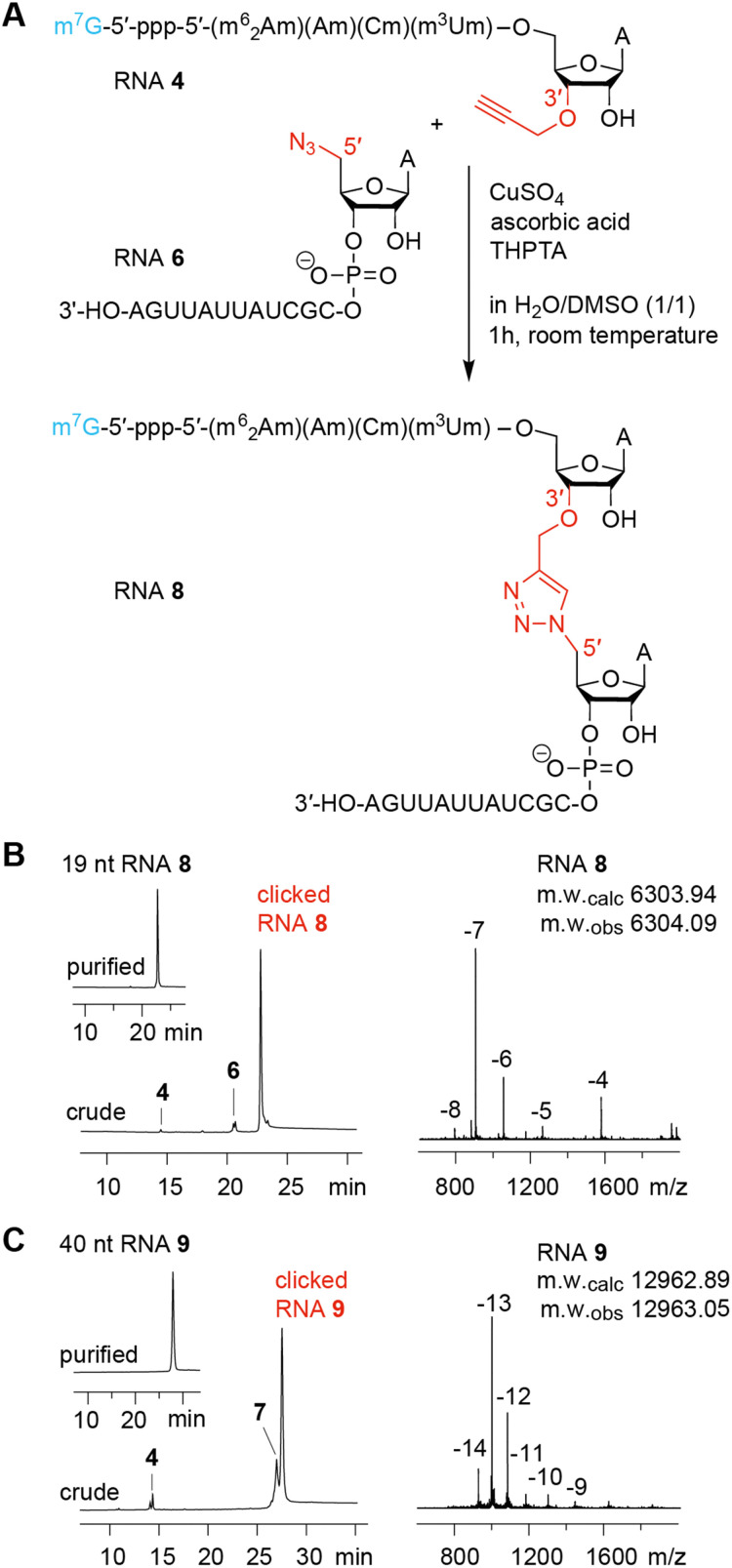
Chemical ligation using CuAAC reaction of 3′-alkyne modified cap-RNA fragments and 5′-azide modified RNA fragments. (A) Exemplary reaction scheme for a 19 nt cap4 RNA. (B) AE HPLC traces for the click ligation of the 19 nt cap4 RNA (left). The crude trace shows the reaction mixture after 1 h at room temperature; the upper left inset displays the purified RNA product; ESI mass spectrum of the product RNA (right). (C) Same as B but for a 40 nt cap4 RNA.

Having the capped RNA-3′-alkynes and 5′-azide-RNAs in our hands, we set out to ligate these fragments according to the original plan. Fig. 5A illustrates a typical CuAAC reaction setup with CuSO_4_, ascorbic acid, and water-soluble tris(3-hydroxypropyltriazolylmethyl)-amine (THPTA) as Cu^I^ stabilizing ligand to react the 3′-alkyne-functionalized m^7^Gppp-hexanucleotide (RNA 4) with the 13 nt RNA 6 providing the 5′ azido group. The use of denaturation conditions (50% dimethyl sulfoxide in H_2_O) removes the need for a ligation template and simplifies the system.^30^ The click ligation proceeded in 1 h at room temperature with almost complete conversion of substrates to the triazole-linked cap4 mimic as reflected in the corresponding AE-HPLC chromatogram of the reaction mixture (Fig. 5B). After HPLC purification, the integrity of the triazole-linked 19 nt long cap4-RNA analog (RNA 8) was confirmed by LC-ESI mass spectrometry (Table 1). Also for longer cap4-RNA target sequences the CuAAC RNA ligation work satisfyingly. After 1 h of incuabtion of RNA 4 and RNA 7 about 80% product yield was cleanly formed (Fig. 5C). The resulting 40 nt RNA (RNA 9) with triazole backbone at the site of ligation was isolated by AE HPLC and the expected molecular weight confirmed by ESI mass spectrometry (Table 1).

To push the limits of the approach we approached 61 and 81 nt cap4 RNA targets, again based on the ligation of the short 6 nt 5′-fragment of m^7^G-5′-ppp-5′-(m_2_^6^Am)(Am)(Cm)(m^3^Um)(3′-*O*-propargyl-A) and synthetic 55 and 75 nt 3′-azide RNA fragments, respectively (Fig. S3, ESI†). Also in this case we obtained significant amounts of ligation products, however, AE HPLC encountered limitation with respect to separation of unreacted RNA from the corresponding cap4-RNA (Fig. S3, ESI†). Nevertheless, the correct molecular weight of the product could be detected unequivocally (Fig. S3, ESI†).

## Conclusions

The azide–alkyne cycloaddition is an immensely powerful technique for labeling and bioconjugation of nucleic acids due to the facile formation, high chemical stability, and compatibility of triazole linkages in cellular systems.^8,10^ More specifically, several examples of artificial triazole backbones, that can mimic natural phosphodiester linkages have been reported so far.^8,10,29^ For our studies, we chose a triazole linkage presented in Fig. 1 and 5A, due to its compatibility with DNA and RNA polymerases.^29,34–36^ Brown *et al.* reported that such a triazole backbone can be also tolerated in functionally critical regions of the single guide (sg) RNA and enable effective Cas9-mediated DNA cleavage *in vitro* and in cells with no unexpected off-target effects.^30^ Moreover, this linkage has the synthetic advantage of being efficiently formed from 5′-azido- and 3′-alkyne-modified oligonucleotides that are readily accessible by the standard RNA synthesis using phosphoramidite chemistry.

The strategy outlined herein represents a significant advancement towards achieving efficient all-chemical synthesis of messenger RNAs (mRNAs) without resorting to *in vitro* transcription (IVT) methods for RNA production or enzymatic ligation of RNA fragments. IVT-based RNA synthesis typically yields a substantial fraction of uncapped transcripts and unwanted double-stranded RNA byproducts, triggering undesired immunogenic responses.^2^ This also necessitates rigorous purification procedures.^2,12^

Further, we point out that our strategy retains the *native* structure of the cap including the first four frequently modified nucleosides; the artificial linkage is located just downstream from that sequence motif that appears critical for recognition of the native capping enzyme machinery and hence the functionality of the cap. This aspect differs to other studies that contain the triazole linkage within the first 5 nucleosides of capped RNA.^8^

Finally, it should be noted that the mRNA targets synthesised here were selected with structural biology applications in mind;^37^ however, we believe that these triazole-linked RNAs could be active in translation. This is suggested by the findings of Hiroyuki Isobe and coworkers on a closely related type of triazole-linked RNA.^38^ Nevertheless, more extensive studies are needed to unlock the full potential of triazole-linked mRNAs for applications in cellular translation.

## Conflicts of interest

There are no conflicts to declare.

## Supplementary Material

CB-005-D4CB00165F-s001

## Data Availability

The data supporting this article have been included as part of the ESI.†
