# Supplementary material for: Access to capped RNAs by chemical ligation
Source: RSC Chem Biol. 2024 Sep 13;5(11):1104–10. doi: 10.1039/d4cb00165f (PMC11393730; doi:10.1039/d4cb00165f)
Supplement: CB-005-D4CB00165F-s001 [file CB-005-D4CB00165F-s001.pdf]

Supplementary Information (SI) for RSC Chemical Biology.
This journal is © The Royal Society of Chemistry 2024

Supporting Information

Access to capped RNAs by chemical ligation

Karolina Bartosik[a] and Ronald Micura*[a]

Contents

Supporting Methods

Synthesis and characterization of guanosine 5′-diphosphates 2
Synthesis of guanosine 5′-diphosphate di-tributylammonium salt 2
Synthesis of guanosine 5′-diphosphate di-triethylammonium salt 2
Synthesis of 7-methylguanosine 5′-diphosphate di-triethylammonium salt 3
Synthesis of 7-methylguanosine 5′-diphosphate imidazolide (Im-m^{7}GDP) 3

RNA solid-phase synthesis 3
Deprotection, purification and quantification of RNAs 4
Mass spectrometry of oligoribonucleotides 5

Synthesis of m^{7}Gppp-RNA 3′-alkyne (RNA **4**) (ROUTE 1) 5
Solid-phase synthesis of Gppp-RNA 3′-alkyne (RNA **3**) 5
Enzymatic N7 methylation of Gppp-RNA 3′-alkyne 6

Synthesis of m^{7}Gppp-RNA 3′-alkyne (RNA **4**) (ROUTE 2) 6
Synthesis of 5′-phosphate RNA 3′-alkyne (RNA **5**) 6
Capping reaction of 5′-p RNA 3′-alkyne with Im-m^{7}GDP 6

Synthesis of 5′-azide RNA (RNA **6**, **7**, **10** and **11**) 7

Non-templated CuAAC ligation: synthesis of RNA **8, 9, 12 and 13** 7

Supporting Figures

Supporting Fig. S1. Characterization data of RNA 3′-alkynes 8

Supporting Fig. S2. Characterization data of 5′-azide RNAs 9

Supporting Fig. S3. Characterization data of 5′-azide RNAs and ligated capped RNAs 10

Supporting Methods

Synthesis and characterization of guanosine 5′-diphosphates

Reagents were purchased from commercial suppliers (Merck/Sigma-Aldrich and CarboSynth) and used without further purification. All reactions were carried out under argon atmosphere. ^{1}H and ^{31}P NMR spectra were recorded on a Bruker Ultrashield^{TM} 400 Plus spectrometer. Chemical shifts (δ) are reported relative to tetramethylsilane (TMS), referenced to the residual solvent signal (D_2O: 4.79 ppm for ^{1}H); ^{31}P shifts are relative to external 85% phosphoric acid. The following abbreviations were used to denote multiplicities: s = singulet, d = doublet, t = triplet, q = quadruplet, m = multiplet. Signal assignments are based on ^{1}H–^{1}H-COSY experiments.

Synthesis of guanosine 5′-diphosphate di-tributylammonium salt

Guanosine 5′-diphosphate di-tributylammonium salt was prepared from the commercially available guanosine 5′-diphosphate disodium salt purchased from CarboSynth. GDP disodium salt (0.5 g, 1.0 mmol, 1.0 equiv.) was dissolved in water (25 mL), and the solution was passed through a glass column filled with 20 mL of wet DOWEX-50WX8 resin 200-400. The filtrate was collected in a flask containing absolute ethanol (12 mL) and tributylamine (0.48 mL, 2.0 mmol, 2.0 equiv.), and stirred at 0°C. The Dowex column was rinsed with water (50 mL) to reach pH 5-6. The solvents were evaporated from the collected solution, and the residue was co-evaporated three times with absolute ethanol and lyophilized from water to yield the product as a white solid. Yield: 765 mg (94%).

^{1}H NMR (400 MHz, D_2O): δ 0.93 (t, J = 7.4 Hz, 18H, CH_3), 1.32-1.42 (m, 12H, CH_2-CH_3), 1.63-1.70 (m, 12H, N-CH_2-CH_2), 3.11-3.15 (m, 12H, N-CH_2), 4.20-4.22 (m, 2H, H_2-C(5')), 4.34-4.37 (m, 1H, H-C(4')), 4.53-4.55 (m, 1H, H-C(3')), 4.73-4.76 (m, 1H, H-C(2')), 5.92 (d, J = 6.0 Hz, 1H, H-C(1')), 8.14 (s, 1H, H-C(8)). ^{31}P NMR (162 MHz, D_2O): δ -11.28 (d), -10.90 (d).

Synthesis of guanosine 5′-diphosphate di-triethylammonium salt

This compound was synthesized as described for guanosine 5′-diphosphate di-tributylammonium salt starting from guanosine 5'-diphosphate disodium salt (0.5 g, 1.0 mmol, 1.0 equiv.) and triethylamine (0.28 mL, 2.0 mmol, 2.0 equiv). Yield: 618 mg (93%).

^{1}H NMR (400 MHz, D_2O): δ 1.28 (t, J = 7.4 Hz, 18H, CH_3), 3.20 (q, J = 7.4 Hz, 12H, CH_2-CH_3), 4.20-4.22 (m, 2H, H_2-C(5')), 4.34-4.37 (m, 1H, H-C(4')), 4.53-4.55 (m, 1H, H-C(3')), 4.72-4.76 (m, 1H, H-C(2')), 5.92 (d, J = 6.0 Hz, 1H, H-C(1')), 8.16 (s, 1H, H-C(8)). ^{31}P NMR (162 MHz, D_2O): δ -11.26 (d), -10.90 (d).

Synthesis of 7-methylguanosine 5′-diphosphate di-triethylammonium salt

To a solution of guanosine 5′-diphosphate di-triethylammonium salt (100 mg, 0.15 mmol, 1.0 equiv.) in anhydrous dimethyl sulfoxide (330 µL), iodomethane (277 µL, 4.5 mmol, 30.0 equiv.) was added and the reaction mixture was stirred under argon atmosphere at room temperature for 3 h. Then the solution was diluted with water (50 mL) and washed with chloroform (50 mL × 5). The aqueous phase was concentrated under reduced pressure, and the product was precipitated with dry acetone (50 mL). After cooling at 4 °C, the precipitate was filtered, washed with cold, dry acetone, and dried *in vacuo*. Crude product was purified by column chromatography on an ÄKTAprime plus system equipped with a LiChroprep® RP-18 column (40-63 µm, 310-25 mm) using 0-15% B in 90 min as a gradient with a flow rate of 4 mL/min (eluent A: 0.1 M TEAB, pH 7.4; eluent B: acetonitrile). The fractions containing the target compound were concentrated, co-evaporated three times with ethanol and dried *in vacuo*. The product was obtained as a white foam. Yield: 42 mg (42%).

^{1}H NMR (400 MHz, D_2O): δ 1.29 (t, *J* = 7.2 Hz, 18H, CH_3), 3.21 (q, *J* = 7.2 Hz, 12H, CH_2-CH_3), 4.13 (s, 3H, *N*-CH_3), 4.22-4.27 (m, 1H, *H*-C(5')), 4.32-4.37 (m, 1H, *H*-C(5')), 4.39-4.42 (m, 1H, *H*-C(4')), 4.54-4.57 (m, 1H, *H*-C(3')), 4.68-4.70 (m, 1H, *H*-C(2')), 6.07 (d, *J* = 3.6 Hz, 1H, *H*-C(1')), 9.25 (s, 1H, *H*-C(8)). ^{31}P NMR (162 MHz, D_2O): δ -11.28 (d), -8.96 (d).

Synthesis of 7-methylguanosine 5′-diphosphate imidazolide (Im-m^{7}GDP)

7-Methylguanosine 5′-diphosphate di-triethylammonium salt (15 mg, 0.02 mmol, 1.0 equiv.), imidazole (11 mg, 0.16 mmol, 8.0 equiv.) and 2,2'-dithiodipyridine (13 mg, 0.06 mmol, 3.0 equiv.) were dissolved in anhydrous DMF (375 µL). Triethylamine (6 µL, 0.04 mmol, 2.0 equiv.) and triphenylphosphine (16 mg, 0.06 mmol, 3.0 equiv.) were added subsequently, and the mixture was stirred for 24 h at room temperature under an argon atmosphere. The product was precipitated from the reaction mixture with a solution of $NaClO_4$ in dry acetone (50 mM, 1.5 mL). The mixture was cooled at 4 ºC and centrifuged to precipitate the target compound. The precipitate was washed with anhydrous acetone and the target compound was obtained as a slightly yellowish solid. Yield: (9 mg, 82%).

^{1}H NMR (400 MHz, D_2O): δ 4.09 (s, 3H, *N*-CH_3), 4.23-4.28 (m, 1H, *H*-C(5')), 4.35-4.37 (m, 1H, *H*-C(5')), 4.38-4.41 (m, 1H, *H*-C(4')), 4.63-4.65 (m, 1H, *H*-C(3')), 4.68-4.73 (m, 1H, *H*-C(2')), 6.05 (d, *J* = 3.6 Hz, 1H, *H*-C(1')), 7.09 (s, 1H, *H*-C(4)^{Im}), 7.37 (s, 1H, *H*-C(5)^{Im}), 8.03 (s, 1H, *H*-C(2)^{Im}). ^{31}P NMR (162 MHz, D_2O): δ -20.05 (d), -11.97 (d).

RNA solid-phase synthesis

RNA synthesis was performed on an H6 GeneWorld DNA/RNA automated synthesizer (K&A, Laborgeraete GbR, Germany) on a 1.0 µmol scale using a standard phosphoramidite chemistry. 2'-*O*-silyl and acetyl protected canonical nucleoside building blocks, 2'-*O*-methyl

adenosine (*N*-Bz) and 2′-*O*-methyl cytidine (*N*-Ac) phosphoramidites as well as CPG solid supports (2′-*O*-TBS rA(pac) 3'-lcaa CPG 1000 Å, 2′-*O*-TBS rG(pac) 3′-lcaa CPG 1000 Å and 3′-*O*-propargyl A(Bz) 2'-lcaa CPG 1000 Å) were purchased from ChemGenes. Phosphoramidites of 3-*N*-methyl-2′-*O*-methyluridine and 6-(*N,N*-dimethyl)-2′-*O*-methyladenosine were prepared according to Leiter, D. Reichert, A. Rentmeister and R. Micura, *ChemBioChem*, **2020**, *21*, 265–271. Oligoribonucleotides were phosphorylated at the 5′ ends on the synthesizer using 3-DMTrO-2,2-bis(ethoxycarbonyl)propyl 2-cyanoethyl *N*,*N*-diisopropylphosphoramidite. Detritylation, coupling, capping and oxidation reagents were dichloroacetic acid/1,2-dichloroethane (4/96), phosphoramidite/acetonitrile (100 mM) and benzylthiotetrazole/acetonitrile (300 mM), Cap A/Cap B (1/1) (Cap A: 4-(dimethylamino)pyridine/acetonitrile (500 mM), Cap B: acetic anhydride/*sym*-collidine/acetonitrile (2/3/5)) and iodine (20 mM) in tetrahydrofuran/pyridine/H_2O (35/10/5), respectively. Solutions of phosphoramidites and tetrazole were dried over activated molecular sieves (3 Å) overnight.

Deprotection, purification and quantification of RNAs

For deprotection of RNAs the solid support was mixed with aqueous methylamine (40%, 0.50 mL) and aqueous ammonia (28%, 0.50 mL) for 4 h at 40 °C. The supernatant was removed and the solid support was washed three times with H_2O/ethanol (1.0 mL; 1/1). Combined supernatant and washings were evaporated to dryness and the residue was dissolved in a solution of tetrabutylammonium fluoride in tetrahydrofuran (1.0 M, 1.5 mL) and incubated for 16 h at 37 °C for removal of 2′-*O*-silyl protecting groups. The reaction was quenched by addition of triethylammonium acetate/H_2O (1.0 M, 1.5 mL, pH 7.4). Tetrahydrofuran was removed under reduced pressure and the sample was desalted with size-exclusion column chromatography (GE Healthcare, HiPrep™ 26/10 Desalting; Sephadex G25) eluting with H_2O; collected fractions were evaporated and the RNA dissolved in H_2O (1 mL).

For deprotection of Gppp-RNA, the solid support in the synthesis cartridge was pre-treated with 1,8-diazabicycloundec-7-en (DBU) in acetonitrile (1.0 M, 0.5 mL) for 5 min at room temperature, then washed with acetonitrile and dried. The CPG beads were transferred to a screw-capped vial and the deprotection was continued according to the procedure described above.

The crude RNA was purified by anion exchange chromatography (Thermo Scientific Ultimate 3000 HPLC System) on a semipreparative Dionex DNAPac® PA-100 column (9 mm × 250 mm) at 80 °C with a flow rate of 2 mL/min (eluent A: 20 mM $NaClO_4$ and 25 mM Tris·HCl (pH 8.0) in 20% aqueous acetonitrile; eluent B: 0.6 M $NaClO_4$ and 25 mM Tris·HCl (pH 8.0) in 20% aqueous acetonitrile). Fractions containing RNA were evaporated and the residue redissolved in 0.1 M triethylammonium bicarbonate solution (10 to 20 mL), loaded on a C18

SepPak Plus® cartridge (Waters/Millipore), washed with 10 mM TEAB solution (for RNAs up to 6 nt) or H_2O (for longer RNAs), and then eluted with acetonitrile/H_2O (1/1).

Crude and purified RNAs were analyzed by anion exchange chromatography (Thermo Scientific Ultimate 3000 HPLC System) on a Dionex DNAPac® PA-100 column (4 mm × 250 mm) at 80 °C with a flow rate of 1 mL/min. For RNAs up to 40 nucleotides in length, a gradient of 0-60% B in 45 min was applied; eluent A: 20 mM $NaClO_4$ and 25 mM Tris·HCl (pH 8.0) in 20% aqueous acetonitrile; eluent B: 0.6 M $NaClO_4$ and 25 mM Tris·HCl (pH 8.0) in 20% aqueous acetonitrile. HPLC traces were recorded at UV absorption by 260 nm. RNA quantification was performed on an Implen P300 Nanophotometer.

Mass spectrometry of oligoribonucleotides

RNA samples (*ca.* 200 pmol) were diluted with aqueous solution of ethylenediaminetetraacetic acid disodium salt dihydrate (Na_2H_2EDTA) (40 mM, 15 µL). Water was added to obtain a total volume of 30 µL. The sample was injected onto a C18 XBridge column (2.5 µm, 2.1 mm × 50 mm) at a flow rate of 0.1 mL/min and eluted using gradient 0 to 100% B at 30 °C (eluent A: 8.6 mM triethylamine, 100 mM 1,1,3,3,3-hexafluoroisopropanol in H_2O; eluent B: methanol). RNA was detected by a Finnigan LCQ Advantage Max electrospray ionization mass spectrometer with 4.0 kV spray voltage in negative mode.

Synthesis of m^7Gppp-RNA 3′-alkyne (RNA 4) (Route 1)

Solid-phase synthesis of Gppp-RNA 3′-alkyne (RNA 3)

The synthesis of Gppp-RNA 3′-alkyne included four steps which were performed in the synthesis cartridge. For each step, the CPG support was treated with the appropriate reagent and shaken at room temperature for the indicated time. For phosphitylation, oxidation and Gpp attachment, a few beads of activated 3 Å molecular sieves were added to the syringe.

The fully protected resin-bound 5′-OH RNA 3'-alkyne (1.0 µmol) was treated with 0.5 mL of phosphitylation solution (0.5 mL of diphenyl phosphite, 2.0 mL of anhydrous pyridine), which was manually passed through the column (using plastic syringes) and allowed to react for 10 min. After washing the beads with ACN, 0.5 mL of hydrolysis solution (0.5 mL of 1 M aqueous triethylammonium bicarbonate, 2.5 mL of H_2O and 2.0 mL of ACN) was applied for 20 min. The solid support was then washed with ACN and dried *in vacuo* for 2 h. Then, the CPG beads were treated with 0.5 mL of oxidation solution (300 mg of imidazole, 1.0 mL of *N,O*-bis(trimethylsilyl)acetamide, 2.0 mL of anhydrous acetonitrile, 2.0 mL of bromotrichloromethane and 0.2 mL of triethylamine) for 1 h. The solution was removed, and the support was washed with ACN. Finally, Gpp attachment was achieved by applying of 0.5

mL of coupling solution (0.28 M guanosine 5′-diphosphate di-tributylammonium salt in dry DMF and 500 mM of $ZnCl_2$) for 16 h. The solution was removed, and the support was washed with H_2O (2 × 0.5 mL), 40 mM aqueous solution of EDTA (2 × 0.5 mL) and ACN (3 × 0.5 mL) and then dried *in vacuo*. RNA was cleaved from the support, deprotected and purified by AE HPLC. The final product was confirmed by LC-ESI mass spectrometry (Supporting Figure 1A).

Enzymatic N7 methylation of Gppp-RNA 3′-alkyne

Lyophilized Gppp-RNA 3′-alkyne (20 nmol, 1.0 equiv) was dissolved in a buffer (40 µL, 1.5 M NaCl, 200 mM Na_2HPO_4, pH 7.4) followed by the addition of an aqueous solution of S-adenosylmethionine (90 µL, 90 nmol, 4.5 equiv), and the addition of water to obtain a total volume of 344 µL. To the mixed solution, 8 µL of 50 µM MTAN (5’-methylthioadenosine/*S*-adenosylhomocysteine nucleosidase), 8 µL of 50 µM LuxS (*S*-ribosyl homocysteine lyase) and 40 µL of 50 µM Ecm1 (*Encephalitozoon cuniculi* mRNA cap (guanine N7) methyltransferase) were added sequentially; enzymes were provided by the group of Andrea Rentmeister, University of Munich, Germany. After incubation at 37 °C for 1 h, the reaction mixture was stopped by phenol/chloroform extraction. Analysis of the reaction and purification of the product were performed by AE chromatography. The final product was confirmed by LC-ESI mass spectrometry (Supporting Figure 1A).

Synthesis of m^7Gppp-RNA 3′-alkyne (RNA 4) (Route 2)

Synthesis of 5′-phosphate RNA 3′-alkyne (RNA 5)

The synthesis of 5′-phosphate RNA 3′-alkyne was performed on 3′-*O*-propargyl A(Bz) CPG support according to the standard procedure. After the synthesis, RNA was cleaved from the support, deprotected and purified by AE HPLC. The final product was confirmed by LC-ESI mass spectrometry (Supporting Figure 1B).

Capping reaction of 5′-p RNA 3′-alkyne with Im-m^7GDP

Triethylammonium salt of fully deprotected 5′-p RNA 3′-alkyne (20 nmol, 1.0 equiv) was dissolved in DMSO (175 µL) and a solution of *P*-imidazolide of *N*7-methylguanosine 5′-diphosphate [Im-m^7GDP] in DMSO (200 µL, 1.0 µmol, 50.0 equiv) and a solution of $ZnCl_2$ in DMSO (25 µL, 0.5 µmol, 25.0 equiv) were added. The mixture was stirred at room temperature for 24 h or at 55 °C for 3 h and the reaction was quenched by addition of 250 µL of aqueous solution of EDTA (20 mg/mL) and $NaHCO_3$ (10 mg/mL). The product was desalted with size-exclusion column chromatography (GE Healthcare, HiPrep™ 26/10 Desalting; Sephadex G25) eluting with H_2O; collected fractions were evaporated and the RNA dissolved in H_2O (1 mL).

Analysis of the reaction and purification of the product were performed by AE chromatography. The final product was confirmed by LC-ESI mass spectrometry (Supporting Figure 1B).

Synthesis of 5′-azide RNA (RNA 6, 7, 10 and 11)

After automated solid-phase synthesis, the fully protected resin-bound 5′-OH RNA (0.5 µmol) was treated with methyltriphenoxyphosphonium iodide (MTPI) in anhydrous DMF (0.5 M, 0.5 mL) for 1 h at room temperature. The solid support was washed with dry DMF (3 × 200 µL) and dried *in vacuo*. A saturated sodium azide solution was then prepared by heating sodium azide (25 mg) resuspended in dry DMF (0.5 mL) for 10 min at 70 °C. After cooling to room temperature, the resin was treated with the solution of azide for 5 h at 55 °C. The resin was then washed with DMF (3 × 200 µL), dry ACN (3 × 200 µL) and dried *in vacuo*. RNA was cleaved from the support, deprotected and purified by AE HPLC (Supporting Figure 2, 3). The final product was confirmed by LC-ESI mass spectrometry (Supporting Figure 2, 3).

Non-templated CuAAC ligation: synthesis of RNA 8, 9, 12 and 13

m^7G-ppp-RNA 3′-alkyne (5.0 nmol in 5 µL H_2O) and 5′-azido-RNA (6.0 nmol in 6 µL H_2O) were mixed with $MgCl_2$ (100 mM, 5 µL), triethylammonium acetate buffer (1 M, pH 7, 20 µL), DMSO (49 µL) and fresh ascorbic acid (125 mM, 10 µL). While degassing the oligonucleotide solution with argon, a solution of $CuSO_4$-tris(3-hydroxypropyltriazolylmethyl) amine (250 mM in 55% v/v DMSO to H_2O, 5 µL) was added and the reaction (final volume = 100 µL) was left for 1 h at room temperature. Then, sodium acetate (3.0 M, 20 µL) and ethanol (300 µL) were added and the RNA was stored at 4 °C for 2 h. After centrifugation (13000 rpm, 20 min, 4 °C), the supernatant was discarded and the pellet was washed twice with ethanol (100 µL). The pellet was then dried *in vacuo* and dissolved in H_2O. Analysis of the reaction and purification of the product were performed by AE chromatography. The final product was confirmed by LC-ESI mass spectrometry.

Supporting Figures

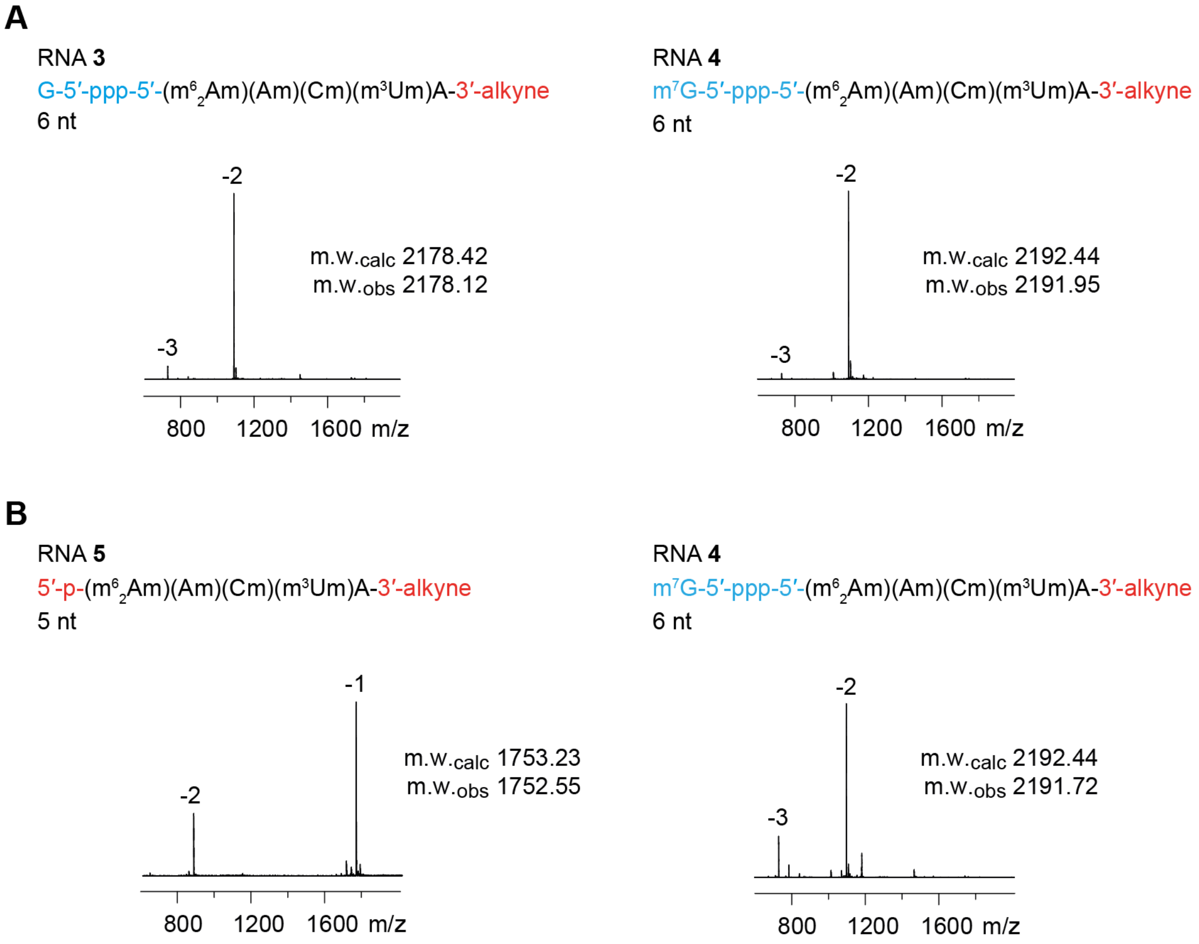

Supporting Figure 1. Characterization data of RNA 3′-alkynes. (**A**) LC-ESI mass spectra of Gppp-RNA 3′-alkyne (RNA **3**) and m^7Gppp-RNA 3′-alkyne (RNA **4**) synthesized according to the ROUTE 1; (B) LC-ESI mass spectra of 5′-phosphate RNA 3′-alkyne (RNA **5**) and m^7Gppp-RNA 3′-alkyne (RNA **4**) synthesized according to the ROUTE 2.

RNA **6**
5′-N_3-ACGCUAUUAUUGA-OH-3′
13 nt

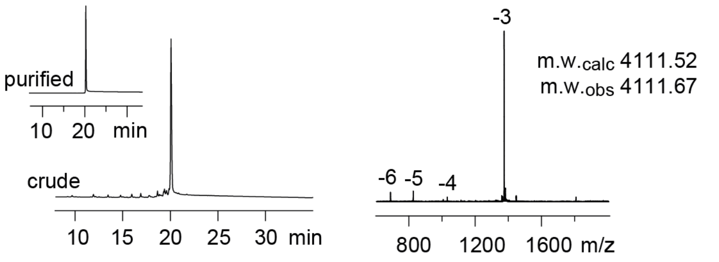

RNA **7**
5′-N_3-ACGCUAUUAUUGAUACA-
GUUUCUGUACUAUAUUG-OH-3′
34 nt

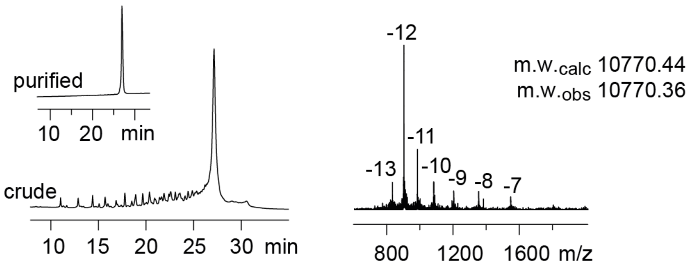

Supporting Figure 2. Characterization data of 5′-azide RNAs. Sequences (left), anion-exchange HPLC traces (middle) and the corresponding LC-ESI mass spectra (right).

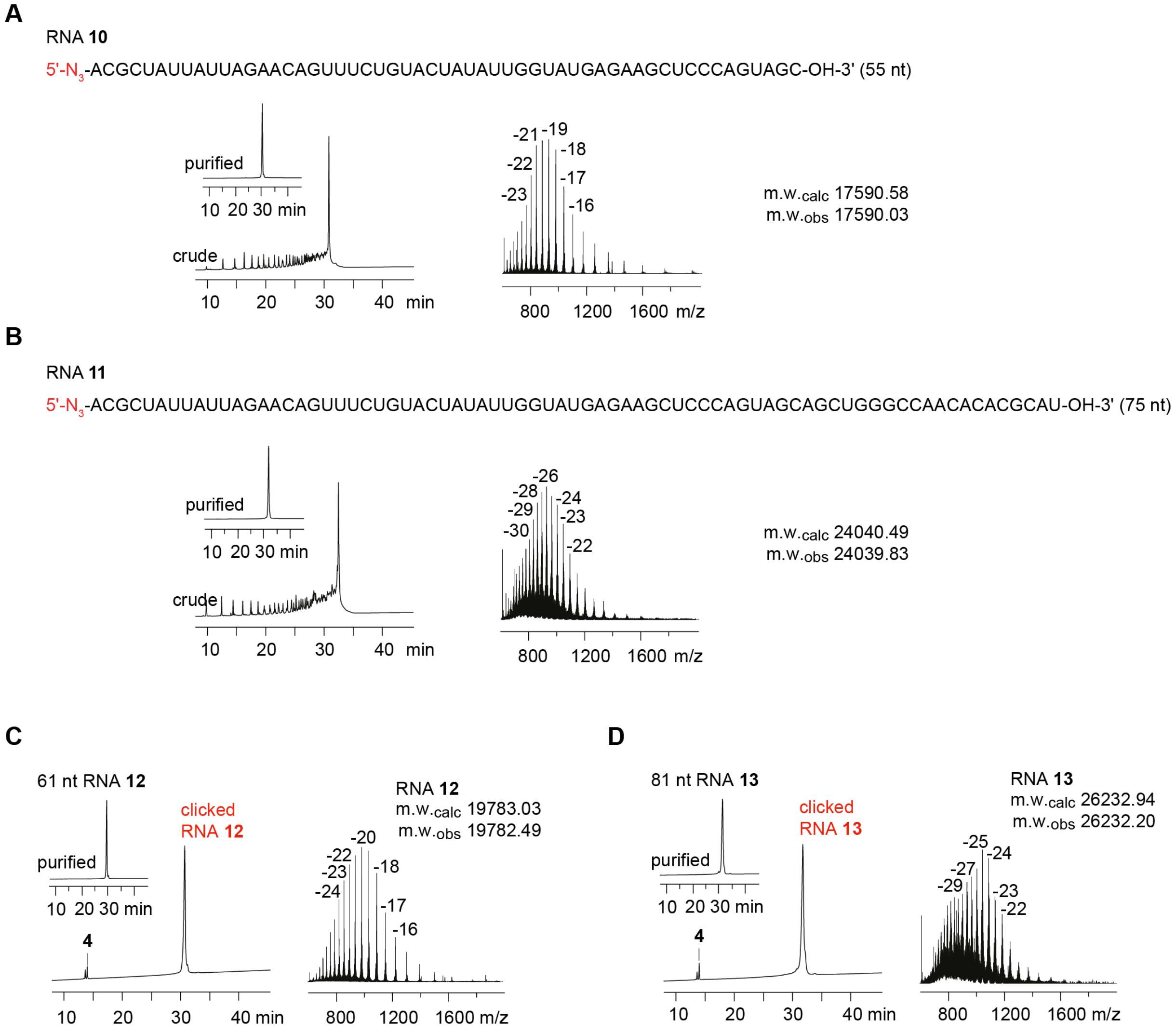

Supporting Figure 3. Characterization data of 5′-azide RNAs and ligated capped RNAs. (**A**, **B**) 5′-azide RNAs: Sequences (top), anion-exchange HPLC traces (left), and LC-ESI mass spectra (right). (**C**) AE HPLC traces for the click ligation of the 61 nt cap4 RNA **12** (left). The crude trace shows the reaction mixture after 1 h at room temperature; the upper left inset displays the purified RNA product; ESI mass spectrum of the product RNA (right). (**C**) Same as (C) but for 81 nt cap4 RNA **13**.
